# Effects of monensin supplementation on lactation performance of dairy cows: a systematic review and dose–response meta‑analysis

**DOI:** 10.1038/s41598-023-27395-9

**Published:** 2023-01-11

**Authors:** M. R. Rezaei Ahvanooei, M. A. Norouzian, A. H. Piray, P. Vahmani, M. H. Ghaffari

**Affiliations:** 1 Department of Animals and Poultry Science, College of Aburaihan, University of Tehran, Tehran, 3391653755 Iran; 2 Department of Animal Science, College of Agriculture and Natural Resources, Razi University, PO Box 6715685418, Kermanshah, Iran; 3 Department of Animal Science, University of California, 2251 Meyer Hall, Davis, CA 95616 USA; 4 Institute of Animal Science, University of Bonn, 53115 Bonn, Germany

**Keywords:** Computational biology and bioinformatics, Literature mining

## Abstract

The aim of this study was to conduct a comprehensive review with meta-analysis to determine the effects of the dose–response relationship between monensin supplementation and dairy cow performance and milk composition. Results from 566 full-text articles and 48 articles with 52 studies were meta-analyzed for pooled estimates. Monensin supplementation up to 23 ppm increased milk production, with the optimal dose being 12.6 ppm. Monensin supplementation at doses ranging from 16 to 96 ppm increased milk production in the prepartum phase (− 28 to 0 day relative to calving). From 60 to 150 DIM, monensin supplementation up to 21 ppm had a significant positive effect on this outcome, while supplementation in the 37 to 96 ppm range caused a decrease in this variable. At 0 to 60 and > 150 DIM, monensin supplementation had no effect on milk yield. At dosages of 22 to 96 ppm, 12 to 36 ppm, and below 58 ppm and 35 ppm, respectively, monensin supplementation resulted in significant decreases in dry matter intake (DMI), milk protein percentage, milk fat percentage, and milk fat yield. Overall, based on the results of this meta-analysis and considering all variables, the recommended optimal dose of monensin could be about 16 ppm.

## Introduction

The inclusion of feed additives such as antibiotics (ionophores or non-ionophores) to the diet to alter the fermentation pattern in the rumen is one of the nutritional strategies used since the 1950s to improve feed efficiency in ruminants^1^. Carboxylic ionophores, including lasalocid, monensin, salinomycin, narasin, and maduramycin, are used as growth stimulants in ruminants, with monensin being the most commonly used agent^2^. Monensin disrupts transmembrane movement and intracellular balance of ions in certain classes of bacteria and protozoa found in the gastrointestinal tract^3^ and triggers a selection mechanism for certain types of microorganisms, which may be beneficial to the host. However, the use of monensin can lead to resistance of certain bacterial strains to various antimicrobial agents, which is a major public health concern^4^. Many studies have shown that monensin supplementation improves dry matter intake^5^ and milk yield^6^, prevents metabolic diseases^7,8^, and reduces methane emissions^9^. However, different optimal monensin doses have been recommended depending on experimental conditions.

Several studies have been published examining the effects of monensin on lactation performance of dairy cows, but results have been inconsistent^10,11^. Several studies found an increase in milk yield^12–14^, but others showed no effect^15,16^. A number of factors can alter the response to monensin on milk production, including herd^17^, BCS^13^, and genetic performance^18,19^. Similar inconsistency has been found in studies of the effects of monensin and dry matter intake (DMI). A meta-analysis is a useful tool to obtain accurate, reliable, and generalizable results in different scientific fields. It provides a comprehensive analysis of the treatment effect, examines possible sources of heterogeneity in the response of animals to an independent variable, and identifies the possible limitations of the study^20^. Two meta-analyzes have been conducted on the effects of monensin on dairy cows^10,11^; both studies used the traditional meta-analysis framework.

In traditional meta-analyzes, correlations between outcomes and doses used in a study are not considered. These correlations are considered in dose–response meta-analysis. In addition, it indicates the optimal level of the independent variable when non-linear relationships exist between outcomes. In addition, Duffield et al.^10^ did not perform a sensitivity analysis for the relevant studies included in the meta-analysis. Therefore, there could be one or more influential studies that could change the model result. In addition, this study did not consider parity as a potential source of heterogeneity. In addition, this meta-analysis was conducted in 2008. Because a substantial number of new studies were published between 2008 and 2021, a new meta-analysis is needed. A recently published meta-analysis by de Moura et al.^11^ is a standard meta-analysis with shortcomings, including: (1) they did not perform a sensitivity analysis and (2) their meta-analysis did not consider parity and year of publication of articles as possible sources of heterogeneity. There are two methods for dose–response meta-analysis, namely the one-stage method and the two-step method. Most previous studies have used the two-stage method. A dose–response meta-analysis using the two-stage approach requires at least three doses of the independent variable in each eligible study. In contrast, the one-stage dose–response meta-analysis includes eligible studies with two doses of the independent variable^21^. In addition, the one-stage approach may provide more accurate insight into the sources of heterogeneity among studies. According to Crippa et al.^21^, the one-stage method can replace the traditional two-stage method in detecting truly curvilinear dose–response relationships. A one-stage dose–response meta-analysis allows the complexity of the research question to be explored by including all eligible studies^21^.

The hypothesis of this study was that monensin supplementation at optimal doses would significantly improve lactation performance in dairy cows. Due to the limitations of previous meta-analyzes and the need to determine the optimal dose of monensin supplementation, the present one-stage dose–response meta-analysis was conducted to investigate the effects of monensin supplementation on feed intake, milk yield and composition, body weight (BW), and body condition score (BCS) in dairy cows.

## Materials and methods

### Data sources

**Figure 1:**
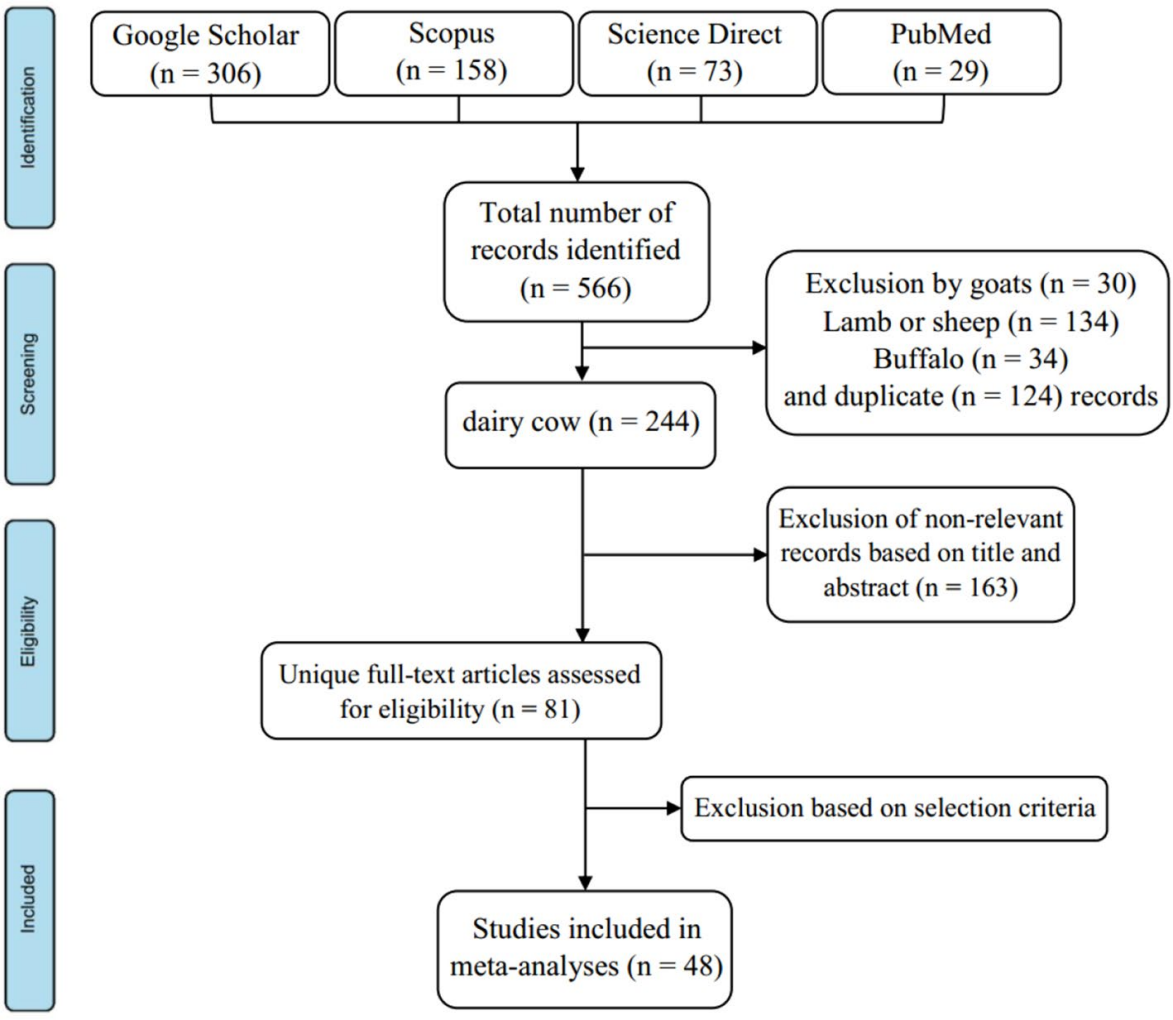
Flowchart of the literature search, identification, and screening process for selecting studies (search conducted from April 2021 to October 2021).

A literature search was performed in several databases, including Google Scholar, Scopus, Science Direct, and PubMed. The keywords used to search the databases were "dairy cow", "dairy cattle", "monensin", "ionophore", "performance", "milk yield", and "milk composition". A total of 566 studies were identified through database searches: Google Scholar (306), Scopus (158), Science Direct (73), and PubMed (29, Fig. 1). The first round of exclusion excluded studies conducted in goats (n = 30), lambs or sheep (n = 134), and buffaloes (n = 34), as well as duplicate studies (n = 124), leaving 244 studies suitable for the meta-analysis. In the second round of screening, 163 irrelevant articles were excluded and 81 articles remained. In these two stages, the title and, if necessary, the abstracts were reviewed. The final screening was performed to select eligible studies according to the following criteria: (1) the study was conducted between 2000 and 2021, (2) the study used a control group without monensin supplementation, (3) the study reported at least one of the outcome variables listed, and (4) the study reported the mean values and associated error. Finally, 48 articles including 52 studies met the required criteria and were included in this meta-analysis. A summary of the details of the articles used in this meta-analysis is provided in Table 1. Outcome measures examined in our meta-analysis included DMI, milk yield, milk fat, milk protein, milk lactose, milk urea nitrogen (MUN), BCS, and BW.

**Table 1:** Summary of studies used in the meta-analysis on the effect of monensin supplementation in dairy cows.

Author	Studies	DM^a^	Method^b^	Diet			TC No.^c^	DIM^d^	ED^e^	Parity	Breed	Outcomes
				Type	CP^ab^ (%)	NDF^ac^ (%)						
Phipps et al. (2000)^14^	1	7.77/15.95/23.8	TMR-top	TMR	19.2	36.1	60	49	140	M	HF^k^	DMI, MY, F, P, L
Ruiz et al. (2001)^52^	1	18.32	TMR	TMR	18.1	51.4	30	126	17	M	H	DMI, MY, F, P, MUN
Vallimont et al. (2001)^53^	1	12.65	TMR-top	TMR	17.8	33.2	60	− 28	92	M	H	DMI, MY, F, P, L, MUN, BCS
Mutsvangwa et al. (2002)^54^	2	14.56/22	CRC/TMR	TMR	17.9	38.7	6	81/150	42	M	H	DMI, MY, F, P, L, MUN
Osborne et al. (2004)^55^	1	22	TMR	TMR	17.7	40.6	6	135	35	M	H	DMI, MY, F, P, L
Erasmus et al. (2005)^56^	1	10	TMR	TMR	18.1	31.2	60	− 21^l^	77	M	HF	DMI, MY, F, P, L, MUN, BW, BCS
Eifert et al. (2005)^57^	1	33	TMR	TMR	18.2	35.9	16	30	84	M	HZ^p^	DMI, MY, F, P, L, BW, BCS
Van Vugt (2005)^58^	2	17.48/18.07	CRC	P + CS/P + WC^q^	7.6/27.2	42.7/27.6	32	150	17	M	HF	DMI, F, P
Bell et al. (2006)^59^	1	24	TMR	TMR	17.3	45.1	28	213	15	P & M	H	DMI, MY, F, P, L
Benchaar et al. (2006)^32^	1	16	TMR	TMR	18.6	35.8	4	98	112	NA	H	DMI, F, P, L, MUN, BW
Odongo et al. (2007)^9^	1	24	TMR	TMR	18.2	34.4	24	92	180	P & M	H	DMI, MY, F, P
Martineau et al. (2007)^60^	1	24	TMR	TMR	17.8	28.9	6	90	105	M	H	DMI, MY, F, P, L, MUN, BW
Yang et al. (2007)^61^	1	16.58	TMR	TMR	15.8	32.2	4	113	84	NA	H	DMI, F, P, L, BW
Karcher et al. (2007)^62^	1	12.5	TMR-top	TMR	13.4	46.7	18	− 28	84	M	H	DMI, MY
Erasmus et al. (2008)^33^	1	15	TMR	TMR	16.6	29.1	40	− 21	81	M	H	DMI, MY, F, P, MUN, BW, BCS
AlZahal et al. (2008)^63^	1	22	TMR	TMR	17.7	35.5	72	138	49	P & M	H	DMI, MY, F, P
Grainger et al. (2008)^64^	1	13.25	CRC	Pasture	16	49	60	46	100	P & M	HF	MY, F, P, L
Chung et al. (2008)^65^	1	15.52	TMR-top	TMR	17.2	33.8	85	− 28	84	M	H	DMI, MY, F, P, L, MUN, BW, BCS
Gehman et al. (2008)^66^	1	14.85	TMR-top	TMR	16.3	33.4	20	101	112	M	H	DMI, MY, F, P, L, MUN, BW, BCS
Martinez et al. (2009)^67^	2	11.07/10.63	TMR-top	TMR	16.9/16.5	31.7/31.4	8	104/139	112	M	H	DMI, MY, F, P, L, MUN, BW
Petit et al. (2009)^68^	1	16	TMR	TMR	14.7	34.3	4	190	112	M	H	DMI, MY, F, P, L, MUN, BW
Fatahnia et al. (2010)^69^	1	10/20/1930	TMR	TMR	15.8	31.9	4	101	84	M	H	DMI, MY, F, P
Gandra et al. (2010)^34^	1	24/48	TMR	TMR	16.77	39.46	12	157	57	NA^m^	H	DMI, F, P, L, BW, BCS
Grainger et al. (2010)^70^	1	23.43	top-dressed	Pasture	18.4	42	20	51	40	M	HF	DMI, MY, F, P, L
Hamilton et al. (2010)^71^	2	21.81/21.35	TMR-top	TMR	17.2	29	18	103	14/60	P	H	DMI, MY, F, P, L, MUN
Baumgard et al. (2011)^72^	1	24.28	TMR-top	TMR^f^	18.8	37.6	36	89	28	M^g^	H^h^	DMI^t^, MY^u^, F^v^, P^w^, L^x^
Mathew et al. (2011)^26^	1	12	TMR	TMR	15.1	37.5	6	194	126	NA	H	DMI, F, P, L, MUN, BW, BCS
He et al. (2012)^73^	1	17.5	TMR	TMR	17	29.3	56	48	147	P & M	H	DMI, MY, F, P, L, MUN, BW, BCS
Gandra et al. (2012)^27^	1	24/48	TMR	TMR	16.8	35	12	157	63	NA	H	DMI, F, P, L, MUN
Abdi et al. (2013)^74^	1	24	TMR	TMR	16.2	35.5	4	83	84	M	H	DMI, MY, F, P, L
Khodamoradi et al. (2013)^75^	1	24	TMR	TMR	16.9	31.6	4	86	84	M	H	DMI, MY, F, P, L
Akins et al. (2014)^35^	1	18	TMR	TMR	18.2	28.4	128	90	28	P^j^ & M	H & HJ^i^	DMI, MY, F, P, L, MUN, BW^z^, BCS^aa^
Rico et al. (2014)^76^	1	16.6	TMR-top	TMR	18.3	28.2	16	183	10	M	H	DMI, MY, F, P
McCarthy et al. (2015)^43^	1	22.5	TMR-top	TMR	13	42.9	70	1	63	P & M	H	DMI, MY, F, P, L, MUN, BW, BCS
Do Prado et al. (2015)^77^	1	16	TMR	TMR	18.8	29.2	4	95	112	M	H	DMI, MY, F, P, L, MUN
Hagen et al. (2015)^78^	1	18	TMR	TMR	16.2	29.5	128	104	70	P & M	H & HJ	DMI, MY, F, P, L, MUN, BW, BCS
Vendramini et al. (2016)^4^	1	24	TMR	TMR	17.26	34.12	24	175	112	M	H	DMI, MY, F, P, L, MUN^y^
de Jesus et al. (2016)^36^	1	22	TMR	TMR	15.7	36.9	24	150	63	M	H	DMI, MY, F, P, L, MUN
Benchaar (2016)^79^	1	24	TMR	TMR	18.2	30.1	8	71	112	M	H	DMI, MY, F, P, L, MUN
Azarfar et al. (2016)^80^	1	24	TMR	TMR	16.9	31.6	4	133	84	M	H	DMI, MY, F, P, L, MUN, BCS
Schären et al. (2017)^81^	1	19.5	CRC^n^	TMR	13.4	37.2	15	− 21	77	P & M	H	DMI, MY, F, P, MUN, BW, BCS
Kozerski et al. (2017)^25^	1	24	TMR	TMR-Pasture	19	59	16	120	28	M	HG^o^	DMI, MY, F, P, L, MUN, BW, BCS
Ghizzi et al. (2018)^5^	1	22	TMR	TMR	16.4	29	36	201	42	M	H	DMI, MY, F, P, L
Santos et al. (2019)^23^	1	12/24/1948	TMR-top	TMR	18.08	34.09	12	135	84	M	H	DMI, MY, F, P, MUN, BW, BCS
Costa et al. (2020)^24^	1	96	Mix in concentrate	P + C^s^	14.6	43.5	8	202	84	M	H	DMI, MY, F, P, L
Grigoletto et al. (2021)^6^	1	20	TMR	TMR	16.9	38.8	40	187	63	NA	H	DMI, F, P, L, MUN
e Silva et al. (2021)^2^	1	24	TMR	TMR	16.4	31.6	8	100	84	NA	J^r^	DMI, F, P, L, MUN, BW, BCS
Vasquez et al. (2021)^51^	1	15.62	TMR	TMR	18.55	37.76	102	− 21	105	P & M	H	DMI, MY, F, P, L, MUN, BW, BCS

^a^Dosage of monensin (mg/kg DM). ^b^Method of monensin supplementation. ^c^Total cows’ number. ^d^Days in milk at treatment start. ^e^Experiment duration (d). ^f^Total mixed rations. ^g^Multiparous. ^h^Holstein. ^i^Holstein and Holstein × Jersey. ^j^Primiparous. ^k^Holstein–Friesian. ^l^The "–" sign on the number indicates the days before calving. ^m^Not available. ^n^Controlled-release capsule. ^o^Holstein-Gyr. ^p^Holstein-Zebu. ^q^Pasture + Corn silage/Pasture + White clover. ^r^Jersey. ^s^Pasture + concentrate. ^t^Dry matter intake. ^u^Milk yield. ^v^Milk fat. ^w^Milk protein. ^x^Milk lactose. ^y^Milk urea nitrogen. ^z^Body weight. ^aa^Body condition score. ^ab^Crude protein. ^ac^Neutral detergent fiber.

### Statistical analysis

In the present study, the mean difference was used as the effect size. Because of the superiority of the one-stage method^22^, the one-stage random-effects meta-analysis approach was used to assess the potential non-linear relationship between dietary monensin supplementation and outcome variables. This was done using a restricted cubic spline with three knots at the fixed percentiles (10th, 50th, and 90th). An overall *P* value was calculated by testing that the two regression coefficients were simultaneously equal to zero. A *P* value of nonlinearity was calculated by testing that the coefficient of the second spline was equal to 0^21^.

Heterogeneity between studies was measured by the variance partition coefficient^21^. When a significant level of heterogeneity was found, subgroup analysis was conducted to examine the potential sources of heterogeneity. Several rules were followed when conducting subgroup analyzes: (1) each subgroup analysis must be based on sound scientific evidence, (2) analyzes were pre-defined, (3) the overall effect of the independent variable was significant, and (4) the subgroup analysis was conducted on data subgroups of the meta-analysis with heterogeneity of > 50% and ≥ 10 studies^22^. Potential factors that may influence dairy cow response to supplemental monensin include parity (multiparous, multiparous and primiparous, and primiparous), decade of article publication (2000–2010 and 2011–2021), the method of using monensin (total mixed ration, TMR), TMR-top dressed, controlled-release capsule, and other), type of diet (TMR, pasture, and others), protein and NDF content of the diet (divided into two categories based on median), number of cows per trial (sample size), number of DIM at the beginning of the trial (day − 28 to 0 (calving), 0 to 60, 60 to 150, and > 150), length of the trial period (divided into two categories based on median), and breed of cows (Holstein, Holstein × Friesian, and others). Publication bias was assessed using Begg's funnel plot and Egger regression asymmetry for meta-analysis datasets with heterogeneity < 50% and ≥ 10 trials^22^.

Sensitivity analysis "leave-one-out approach, location of knots, and removing high monensin supplementation (> 48 ppm)" was performed to evaluate the robustness of the results^22^. In the spline model, the position of the knots may affect the results obtained. Therefore, we tested the sensitivity of the estimated curves to the position of the knots. To do this, we examined alternative knot locations, including various combinations of the 10th, 25th, 50th, 75th, and 90th percentiles of the total dose distributions. The results showed that the estimated curves were not sensitive to the location of the knots. Statistical analyzes were performed using the *dosresmeta* and *metafor* packages in R software (https://www.r-project.org/). A *P* ≤ 0.05 was considered statistically significant.

## Results

### Pre-analysis superficial review

**Figure 2:**
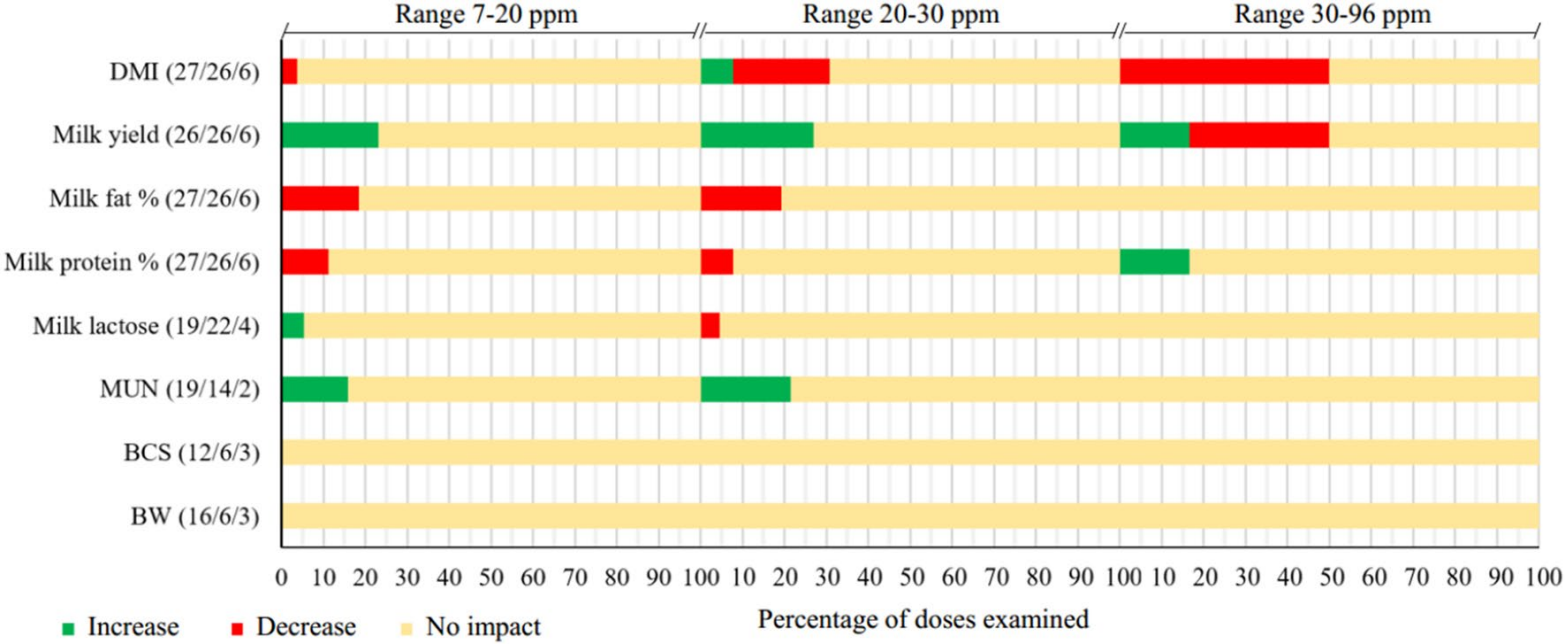
Percent effectiveness of "examined monensin doses" on outcomes in dairy cows. The effect was considered significant if the overall *P* value of the model was less than 0.05. Numbers in parentheses preceding results indicate the number of studies that examined the above dose ranges, separated by the slash '/'.

Figure 2 and Supplemental Table S1 provide general information about the effect of monensin supplementation on outcome variables. The corresponding studies examined various dosages of monensin supplementation ranging from 0 to 96 ppm. The figure also shows that most of the monensin dosages studied had no effect on the outcome variables. Monensin supplementation appeared to have the greatest effect on DMI and milk yield, while other outcomes were less affected.

### Effect on DMI

**Figure 3:**
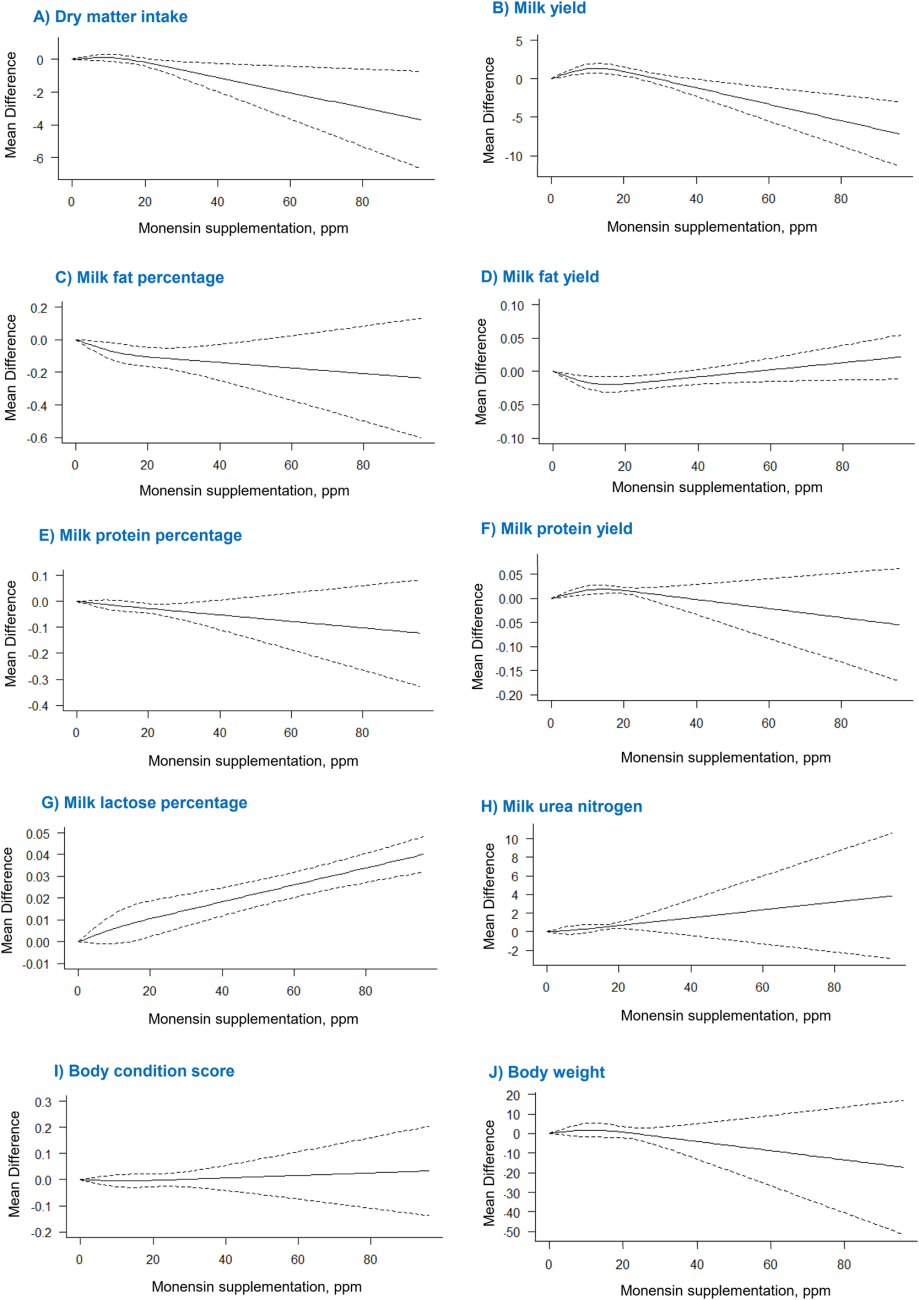
Dose–response association between monensin supplementation and (**A**) dry matter intake (kg/day), (**B**) milk yield (kg/day), (**C**) milk fat percentage, (**D**) milk fat yield (kg/day), (**E**) milk protein percentage, (**F**) milk protein yield (kg/day), (**I**) milk lactose percentage, (**G**) milk urea nitrogen (mg/dL), (**K**) body condition score, and (**L**) body weight (kg). The solid line and the dashed lines represent the estimated mean difference and its 95% confidence intervals.

**Figure 4:**
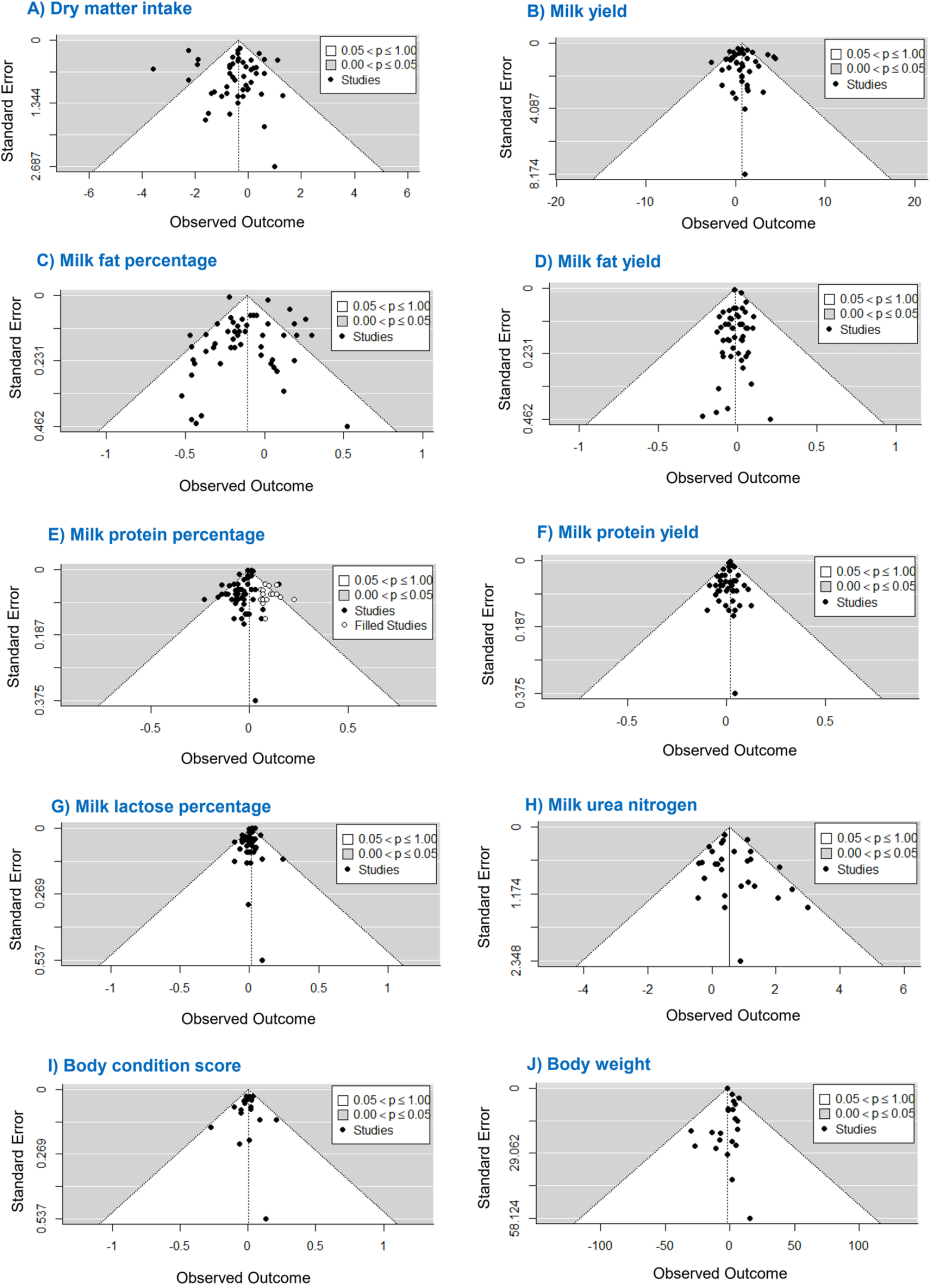
Contour-enhanced funnel plots of studies investigating the association between monensin supplementation and (**A**) dry matter intake (kg/day), (**B**) milk yield (kg/day), (**C**) milk fat percentage, (**D**) milk fat yield (kg/day), (**E**) milk protein percentage, (**F**) milk protein yield (kg/day), (**I**) milk lactose percentage, (**G**) milk urea nitrogen (mg/dL), (**K**) body condition score, and (**L**) body weight (kg). Dotted lines represent 95% pseudo-confidence interval. *SE* standard error.

Forty-seven eligible articles, including 51 studies, were included in the analysis to evaluate the effects of monensin supplementation on DMI. The results showed a significant effect of monensin supplementation on DMI (*P* = 0.04). Dry matter intake was significantly decreased by increasing the monensin dose from 22 to 96 ppm (Fig. 3A). Leave-one-out analysis did not reveal any influential study that changed the results of the model. In addition, the overall trend of the association did not change when the monensin dose was removed above 48 ppm and the location of the knots. These tests of sensitivity analysis indicate that the results are statistically robust. No significant heterogeneity was observed for this outcome measure (heterogeneity < 50%). In addition, publication bias was not significant (*P* > 0.10, Fig. 4A).

### Effect on milk yield

Forty eligible articles, including 43 studies, examined the effects of monensin supplementation on milk yield. A significant non-linear association was found between monensin supplementation and milk yield (*P* < 0.01). Increasing monensin supplementation dose up to 23 ppm linearly increased milk yield, and supplementation from 24 to 38 ppm monensin had no significant effect on this variable. In contrast, monensin supplementation above 38 ppm caused a significant decrease in milk yield (Fig. 3B). The results suggest that the optimal dose of monensin supplementation to maximize milk yield was 12.61 ppm. The sensitivity analysis of "leave one out, remove doses above 48 ppm, and knots location" did not change the model result. There was high heterogeneity among studies in terms of milk yield (heterogeneity > 50%) and therefore, to explore the causes, subgroup analysis was performed for decade of publication, parity, method of using monensin, type of feed, protein and NDF content of the diet, length of experimental period, and breed of cow. Subgroup analysis revealed that of all these potential factors, only the effect of DIM was significant (*P* < 0.001). Accordingly, monensin supplementation at doses ranging from 16 to 96 ppm significantly increased milk yield in the pre-partum phase (− 28 to 0 day relative to calving). From 60 to 150 of DIM, monensin supplementation up to 21 ppm had a significant positive effect on this outcome; dosages of 22 to 36 ppm monensin were not effective, while its supplementation in the 37 to 96 ppm range reduced milk yield. No significant effect of monensin supplementation was observed at 0 to 60 and > 150 DIM. No publication bias was observed for milk yield (*P* > 0.10, Fig. 4B).

### Effect on milk fat percentage and yield

Forty-seven articles with 51 studies were included in the meta-analysis on the effect of monensin on milk fat. A relatively linear association was observed between monensin supplementation and milk fat (*P* = 0.001). The percentage of milk fat decreased significantly when monensin was added to the diet up to 51 ppm, and its effect at the doses from 52 to 96 ppm was not significant (Fig. 3C). A relatively linear association was also found between the addition of monensin and milk fat yield (*P* = 0.009; Fig. 3D). The addition of monensin at doses 21–31 significantly reduced this result, and outside this range the effect of monensin was not significant. No significant heterogeneity (heterogeneity < 50%) and no publication bias (*P* > 0.10) were observed for milk fat percentage (Fig. 4C) and yield (Fig. 4D). Leave-one-out analysis, removal of a high dose of monensin, and knots location did not alter the outcomes of the models with respect to these results.

### Effect on milk protein percentage and yield

Forty-seven articles with 51 studies were included in this meta-analysis. This meta-analysis revealed a relatively linear relationship between monensin supplementation and milk protein content (*P* = 0.01). Monensin supplementation at doses ranging from 12 to 36 ppm resulted in a significant decrease in milk protein percentage (Fig. 3E), and outside this range, the effect was not significant. Leave-one-out analysis did not identify an influential study. Excluding the data point above 48 ppm monensin from the analysis and the location of the knots also did not change the results. Low heterogeneity was found for this result (heterogeneity < 50%). For milk protein percentage, the funnel plot and Egger's regression test indicated significant publication bias (*P* = 0.04), suggesting that the results should be interpreted with caution (Fig. 4E).

A J-shaped relationship was observed between supplemental administration of monensin and milk protein yield (*P* < 0.001; Fig. 3F), and the estimated optimal dose of monensin was 13.50 ppm. Moreover, the effect of monensin was significant up to a dose of about 24 ppm, above which it was not significant. No significant heterogeneity (heterogeneity < 50%) and publication bias (*P* > 0.10) were observed for milk protein yield (Fig. 4F). Based on leave-one-out analysis, study No. 5^23^ was a highly influential study. Their exclusion changed the model result; all doses of monensin addition resulted in increased milk protein yield. Our results also suggest that an 80% dose (13.87 ppm) is sufficient to maximize milk protein yield. However, removal of doses above 48 ppm did not affect the model result for this outcome.

### Effect on milk lactose percentage

Thirty-eight eligible articles with 41 studies were included in this analysis. Although there was a relatively linear relationship between monensin supplementation and milk lactose percentage, only monensin supplementation at doses ranging from 16 to 96 ppm resulted in increase (trend, *P* = 0.06) in milk lactose percentage (Fig. 3G). Based on the results of the leave-one-out analysis, study No. 41^24^ with a dose of 96 ppm monensin was identified as an influential study. When this study was excluded from the analysis, the overall P value of the model changed from < 0.001 to 0.0552, indicating that monensin supplementation tends to increase lactose content (Fig. 3G). Heterogeneity for milk lactose was low (heterogeneity < 50%). No publication bias was detected in this analysis (*P* > 0.10, Fig. 4G).

### Effect on MUN

For MUN, twenty-nine articles with 32 studies were included in the meta-analysis. According to the results, monensin supplementation linearly increased MUN content (*P* < 0.001, Fig. 3H). In the leave-one-out analysis, studies No. 4^23^, 11^25^, 14^26^, and 27^27^ were found to be influential. Exclusion of these studies from the analysis did not change the statistical significance of the original model, but did change the estimated effect sizes. Monensin supplementation at 13 to 30 ppm dose range increased MUN, and other doses of monensin had no significant effect. Because of the lack of studies with doses greater than 48 ppm, the sensitivity analysis for high doses (> 48 ppm) of monensin supplementation was not performed. The heterogeneity index for MUN was low (heterogeneity < 50%), and there was no significant publication bias (*P* > 0.10, Fig. 4H).

### Effect on BCS and BW

Eighteen studies were used for the meta-analysis by BCS. This analysis found no association between additional monensin intake and BCS (Fig. 3I). No influential study was identified in the leave-one-out analysis, indicating the consistency of the results obtained. No study examined the effects of a high dose (> 48 ppm) of monensin on BCS. There was no evidence of significant heterogeneity (heterogeneity < 50%) and publication bias (*P* > 0.10) for this result (Fig. 4I).

Twenty-one articles involving 22 studies were included in the meta-analysis to evaluate the effect of monensin addition on BW. The results showed that the addition of monensin had no significant effect on BW of dairy cows (Fig. 3J). Leave-one-out analysis did not change the estimates. There was no study with a higher dose than 48 ppm, so the sensitivity analysis for high doses of monensin (> 48 ppm) was not performed. No significant heterogeneity (heterogeneity < 50%) and no publication bias (*P* > 0.10) were observed for this result (Fig. 4J).

## Discussion

Monensin is an ionophore produced by *Streptomyces cinnamonensis*^28^. It is often classified as an antibiotic because it interferes with ion transport across cell membranes, resulting in alteration of the rumen bacterial population through selective bacteriostatic action^28^. It has long been known that monensin improves digestive efficiency and energy metabolism by inhibiting Gram-positive bacteria rather than Gram-negative bacteria^29^. This change in rumen bacterial populations has several implications for ruminant metabolism. The ability of the molecule to alter rumen fermentation to increase the production of propionic acid and decrease the molar fractions of butyric acid, acetic acid, and carbon losses in the form of carbon dioxide and methane improves feed efficiency^28,29^.

Monensin supplementation in lactating dairy cows has been studied extensively. However, Arnqvist et al.^30^ noted that each study alone is of little value unless it is compared with similar studies. Meta-analysis involves summarizing the results of appropriate studies on a topic using statistical methods^31^. This meta-analysis summarizes the results of published articles from 2000 to 2021 on the effects of monensin supplementation in lactating dairy cows. The present meta-analysis showed that supplemental administration of monensin altered the performance parameters of dairy cows. There was a J-shaped association between monensin supplementation and milk yield, and supplementation as low as 23 ppm had a significant positive effect on milk yield. Monensin supplementation in the range of 24 to 38 ppm had no statistically significant effect, and milk yield decreased linearly with doses above 38 ppm. The optimal dose was estimated to be 12.61 ppm. A significant degree of heterogeneity was found in this result. Subgroup analysis indicated that DIM could influence the effectiveness of monensin.

A J-shaped relationship was found between monensin supplementation and milk fat percentage. In addition, there was a linear relationship between monensin supplementation and the percentage of milk protein and lactose. Milk fat and protein percentage decreased linearly with monensin addition in the ranges of 0 to 58 and 12 to 36 ppm, respectively. However, as the monensin dose was increased from 16 to 96 ppm, the percentage of milk lactose tended to increase. The results also showed that the addition of monensin resulted in an increase in milk protein yield, while the addition of monensin at a dosage of 21 to 31 ppm reduced milk fat yield. The relationship between monensin addition and DMI was also J-shaped, with monensin addition at doses above 22 ppm reducing DMI.

The literature indicates that dairy cow responses to monensin supplementation were not consistent. Addition of 8 to 48 ppm monensin to the diet of dairy cows resulted in changes in milk yield ranging from − 1.3 to 2.8 kg/day. Addition of monensin at 15 to 18 ppm resulted in maximum milk yield, and cows receiving the highest monensin dose gave the lowest milk yield^9,23,32–36^. Meta-analysis of 77 studies found that supplemental monensin increased milk yield by only 0.7 kg/day^10^.

The effects of monensin supplementation on milk protein percentage and milk yield were also inconsistent in previous studies. According to Akins et al.^35^, the addition of monensin decreased milk protein percentage, but milk protein yield remained unchanged. However, Benchaar et al.^32^ reported that neither milk protein yield nor percentage changed with monensin supplementation. A previous study of 3577 dairy cows showed that supplemental monensin increased milk protein yield while protein percentage remained unchanged^36^. In contrast, the meta-analysis by Duffield et al.^10^ found that the addition of monensin decreased milk protein percentage.

Similar to this study, inclusion of low to moderate levels of monensin in the diets of dairy cows was found to decrease milk fat yield and milk fat percentage in several previous studies^23,32,33,37,38^. Interestingly, milk fat yield remained unchanged at moderate to high monensin inclusion, and milk fat percentage remained unchanged or decreased^23,35,36^. This meta-analysis confirms the results of the meta-analysis by Duffield et al.^10^, who reported that dietary monensin supplementation resulted in a lower milk fat percentage. This decrease in milk fat percentage could be due to the dilution effect resulted from increased milk volume production^32^.

Monensin has been shown to increase the production of propionate, a glucogenic precursor, and therefore may contribute to a reduction in energy loss during feeding, which could increase glucose and lactose synthesis, leading to improved milk yield^9^. In addition, monensin decreased the deamination of amino acids in the rumen, which decreased the NH3 concentration in the rumen and increased the escape and absorption of gluconeogenic amino acids, which in turn increased the availability of glucose for milk production and lactose^39,40^.

Similar to our findings, Ipharraguerre et al.^41^ reported a 1.5% decrease in DMI in 14 ionophore experiments, while Santos et al.^23^ reported an 18% or 3.58 kg/day decrease in DMI in lactating dairy cows supplemented with high levels (48 mg/kg DM) of monensin. The addition of monensin to a TMR diet at concentrations ranging from 8 to 33 ppm also decreased DMI^42^. Conversely, the addition of monensin had no effect on DMI in light lactating dairy cows^32^. However, in early lactating dairy cows, the addition of monensin increased DMI by 5%^43^. Benchaar et al.^32^ and Ipharraguerre et al.^41^ indicate that the effect of monensin on DMI depends on the stage of lactation, the status of energy balances, the level of monensin administration, and the number of animals used in the study.

The findings from our meta-analysis suggest that monensin supplementation could increase milk urea concentration. However, Duffield et al.^44^, reported no effects of monensin supplementation on milk urea in a meta-analysis study. Mullins et al.^45^ also found an increase in MUN in multiparous Holsteins when 400 mg/day monensin was administered through the feed. Mullins et al.^45^ did not provide a clear explanation for the higher concentration of MUN after monensin administration. Mammi et al.^29^ reported higher MUN with monensin supplementation and attributed this to reduced microbial degradation and higher flux of undegraded proteins into the intestine and the resulting higher contribution of dietary AA absorbed from the small intestine to the profiles of milk AA. This explanation was also supported by the higher blood urea levels^43,45^, and lower ammonia levels in the rumen fluid of monensin-treated cows^46^. Feeding monensin to dairy cows has been shown to reduce deamination from 27 to 17 nmol ammonia mg/protein/min^47^. Blood urea nitrogen distributes freely throughout body fluids, including milk. Thus, this proposed mechanism may explain why MUN was higher in cows receiving monensin^45^.

It has been reported that the addition of 27 to 33 ppm monensin to the diet of dairy cows causes an increase in CP digestion in the small intestine by inhibiting the ruminal protein degradability^42,48^. Moreover, McGuffey et al.^3^ found that supplementation with monensin reduced protein degradation, ammonia accumulation, and microbial nitrogen in rumen fluid in vitro. These results indicate that more dietary protein reaches the small intestine and consequently more amino acids are absorbed by the small intestine when dairy cows' diets are supplemented with monensin. The higher rate of AA absorption may contribute to increased gluconeogenesis from nonessential AA, possibly increasing milk protein synthesis, lactose synthesis, and mammary gland efficiency^49^.

Moreover, Mammi et al.^29^ found that urea synthesis in the liver increased with monensin treatment because the lower lipid accumulation in hepatocytes improved liver functionality. Lower triglycerides accumulation in the liver of monensin-treated cows in early and mild lactation has been noted^16,43^. These results are supported by the higher mRNA abundance of carnitine palmitoyl transferase 1 in the liver, the slower accumulation of triglycerides in the liver^45^, and the better conversion rate of propionate to glucose in monensin-supplemented cows^43^.

The results of this meta-analysis showed no significant effect of monensin supplementation on BW and BCS. Consistent with our results, feeding monensin premix did not alter BCS^35,50^. Also, according to Vasquez et al.^51^, neither total BW nor postpartum BCS was affected by diet type or monensin supplementation. However, the meta-analysis conducted by Duffield et al.^10^ found an improvement in BCS and BW with monensin supplementation.

## Conclusions

This comprehensive review and meta-analysis examined the existing literature on the effects of monensin supplementation on performance and milk composition of dairy cows. The results showed that supplementation with monensin up to 23 ppm increased milk production, and the estimated optimal dose of monensin was 12.61 ppm. The response of milk production to monensin supplementation varied according to DIM. In addition, monensin supplementation significantly decreased DMI, milk protein, milk fat, and milk fat yield at doses of 22 to 96 ppm, 12 to 36 ppm, and below 58 ppm and 35 ppm, respectively. All monensin doses increased milk protein yield, and an 80% dose (13.87 ppm) is sufficient to maximize milk protein yield. In addition, supplementation with monensin at doses ranging from 13 to 30 ppm increased MUN. It tended to increase the lactose content of milk and had no effect on BCS and BW. Overall, the optimal dose of monensin could be 16 ppm.

## Supplementary Information

Supplementary Table S1.

## Data Availability

The datasets used and/or analysed during the current study available from the corresponding author on reasonable request.

## References

[1] Beeson WM, Perry T (1952). Balancing the nutritional deficiencies of roughages for beef steers. J. Anim. Sci..

[2] e Silva S (2021). Effects of plant extract supplementations or monensin on nutrient intake, digestibility, ruminal fermentation and metabolism in dairy cows. Anim. Feed Sci. Technol..

[3] McGuffey R, Richardson L, Wilkinson J (2001). Ionophores for dairy cattle: Current status and future outlook. J. Dairy Sci..

[4] Vendramini T (2016). Effects of a blend of essential oils, chitosan or monensin on nutrient intake and digestibility of lactating dairy cows. Anim. Feed Sci. Technol..

[5] Ghizzi LG (2018). Effects of functional oils on ruminal fermentation, rectal temperature, and performance of dairy cows under high temperature humidity index environment. Anim. Feed Sci. Technol..

[6] Grigoletto NT (2021). Effects of a blend of live yeast and organic minerals or monensin on performance of dairy cows during the hot season. J. Dairy Sci..

[7] McCarthy M (2015). Metabolism of early-lactation dairy cows as affected by dietary starch and monensin supplementation. J. Dairy Sci..

[8] McGuffey R (2017). A 100-year review: Metabolic modifiers in dairy cattle nutrition. J. Dairy Sci..

[9] Odongo N (2007). Long-term effects of feeding monensin on methane production in lactating dairy cows. J. Dairy Sci..

[10] Duffield T, Rabiee A, Lean I (2008). A meta-analysis of the impact of monensin in lactating dairy cattle. Part 2. Production effects. J. Dairy Sci..

[11] de Moura DC (2021). Meta-analysis of the effects of ionophores supplementation on dairy cows performance and ruminal fermentation. Livest. Sci..

[12] Hayes D, Pfeiffer D, Williamson N (1996). Effect of intraruminal monensin capsules on reproductive performance and milk production of dairy cows fed pasture. J. Dairy Sci..

[13] Duffield T (1999). Effect of prepartum administration of monensin in a controlled-release capsule on milk production and milk components in early lactation. J. Dairy Sci..

[14] Phipps R (2000). Effect of monensin on milk production of Holstein–Friesian dairy cows. J. Dairy Sci..

[15] Melendez P, Gonzalez G, Benzaquen M, Risco C, Archbald L (2006). The effect of a monensin controlled-release capsule on the incidence of retained fetal membranes, milk yield and reproductive responses in Holstein cows. Theriogenology.

[16] Zahra L (2006). Effects of rumen-protected choline and monensin on milk production and metabolism of periparturient dairy cows. J. Dairy Sci..

[17] Lean I, Curtis M, Dyson R, Lowe B (1994). Effects of sodium monensin on reproductive performance of dairy cattle. I. Effects on conception rates, calving-to-conception intervals, calving-to-heat and milk production in dairy cows. Aust. Vet. J..

[18] Van der Werf J, Jonker L, Oldenbroek J (1998). Effect of monensin on milk production by Holstein and Jersey cows. J. Dairy Sci..

[19] Granzin B, Dryden GM (1999). The effects of monensin on milk production and levels of metabolites in blood and rumen fluid of Holstein–Friesian cows in early lactation. Aust. J. Exp. Agric..

[20] St-Pierre N (2001). Invited review: Integrating quantitative findings from multiple studies using mixed model methodology. J. Dairy Sci..

[21] Crippa A, Discacciati A, Bottai M, Spiegelman D, Orsini N (2019). One-stage dose–response meta-analysis for aggregated data. Stat. Methods Med. Res..

[22] Piray A, Foroutanifar S (2021). Chromium supplementation on the growth performance, carcass traits, blood constituents, and immune competence of broiler chickens under heat stress: A systematic review and dose-response meta-analysis. Biol. Trace Elem. Res..

[23] Santos MCB (2019). Effects of increasing monensin doses on performance of mid-lactating Holstein cows. J. Appl. Anim. Res..

[24] Costa LPM (2020). Combination of pelleting and monensin does not affect antioxidant properties and fatty acids in milk of grazing dairy cows supplemented with a concentrate containing soybean seeds. Trop. Anim. Health Prod..

[25] Kozerski ND, Signoretti RD, Souza JC, Daley VS, Freitas JA (2017). Use of monensin in lactating crossbred dairy cows (Holstein × Gyr) raised on tropical pastures with concentrate supplementation. Anim. Feed Sci. Technol..

[26] Mathew B, Eastridge M, Oelker E, Firkins J, Karnati S (2011). Interactions of monensin with dietary fat and carbohydrate components on ruminal fermentation and production responses by dairy cows. J. Dairy Sci..

[27] Gandra JR, Rennó FP, Freitas Júnior JE, Maturana Filho M, Barletta RV (2012). Nutrients balances and milk fatty acid profile of mid lactation dairy cows supplemented with monensin. Rev. Bras Saúde Prod. Anim..

[28] Duffield T, Rabiee A, Lean I (2008). A meta-analysis of the impact of monensin in lactating dairy cattle. Part 1. Metabolic effects. J. Dairy Sci..

[29] Mammi LM (2021). The use of monensin for ketosis prevention in dairy cows during the transition period: A systematic review. Animals.

[30] Arnqvist G, Wooster D (1995). Meta-analysis: Synthesizing research findings in ecology and evolution. Trends Ecol. Evol..

[31] Viechtbauer W (2010). Conducting meta-analyses in R with the metafor package. J. Stat. Softw..

[32] Benchaar C, Petit H, Berthiaume R, Whyte T, Chouinard P (2006). Effects of addition of essential oils and monensin premix on digestion, ruminal fermentation, milk production, and milk composition in dairy cows. J. Dairy Sci..

[33] Erasmus L, Muya C, Erasmus S, Coertze R, Catton D (2008). Effect of virginiamycin and monensin supplementation on performance of multiparous Holstein cows. Livest. Sci..

[34] Gandra JR (2010). Productive performance and milk protein fraction composition of dairy cows supplemented with sodium monensin. Rev. Bras. Zootecnia.

[35] Akins M, Perfield K, Green H, Bertics S, Shaver R (2014). Effect of monensin in lactating dairy cow diets at 2 starch concentrations. J. Dairy Sci..

[36] de Jesus EF (2016). Influence of a blend of functional oils or monensin on nutrient intake and digestibility, ruminal fermentation and milk production of dairy cows. Anim. Feed Sci. Technol..

[37] Broderick G (2004). Effect of low level monensin supplementation on the production of dairy cows fed alfalfa silage. J. Dairy Sci..

[38] Dubuc J (2010). A field study on the effects of dietary monensin on milk production and milk composition in dairy cows. Can. Vet. J..

[39] Swanepoel N, Robinson P, Erasmus LJ (2016). Rumen microbial protein flow and plasma amino acid concentrations in early lactation multiparity Holstein cows fed commercial rations, and some relationships with dietary nutrients. Livest. Sci..

[40] Doepel L, Hewage I, Lapierre H (2016). Milk protein yield and mammary metabolism are affected by phenylalanine deficiency but not by threonine or tryptophan deficiency. J. Dairy Sci..

[41] Ipharraguerre IR, Clark JH (2003). Usefulness of ionophores for lactating dairy cows: a review. Anim. Feed Sci. Technol..

[42] Plaizier J (2000). Effect of a prepartum administration of monensin in a controlled-release capsule on apparent digestibilities and nitrogen utilization in transition dairy cows. J. Dairy Sci..

[43] McCarthy M, Yasui T, Ryan C, Mechor G, Overton T (2015). Performance of early-lactation dairy cows as affected by dietary starch and monensin supplementation. J. Dairy Sci..

[44] Duffield T, Rabiee A, Lean I (2008). A meta-analysis of the impact of monensin in lactating dairy cattle. Part 3. Health and reproduction. J. Dairy Sci..

[45] Mullins C (2012). Effects of monensin on metabolic parameters, feeding behavior, and productivity of transition dairy cows. J. Dairy Sci..

[46] Mezzetti M (2019). Monensin controlled-release capsule administered in late-pregnancy differentially affects rumination patterns, metabolic status, and cheese-making properties of the milk in primiparous and multiparous cows. Ital. J. Anim. Sci..

[47] Russell JB, Houlihan AJ (2003). Ionophore resistance of ruminal bacteria and its potential impact on human health. FEMS Microbiol. Rev..

[48] Surber L, Bowman J (1998). Monensin effects on digestion of corn or barley high-concentrate diets. J. Anim. Sci..

[49] Duffield T (1998). Effect of prepartum administration of monensin in a controlled-release capsule on postpartum energy indicators in lactating dairy cows. J. Dairy Sci..

[50] Sauer F, Kramer J, Cantwell W (1989). Antiketogenic effects of monensin in early lactation. J. Dairy Sci..

[51] Vasquez J (2021). Effects of prepartum diets varying in dietary energy density and monensin on early-lactation performance in dairy cows. J. Dairy Sci..

[52] Ruiz R (2001). Effect of monensin on the performance and nitrogen utilization of lactating dairy cows consuming fresh forage. J. Dairy Sci..

[53] Vallimont J, Varga G, Arieli A, Cassidy T, Cummins K (2001). Effects of prepartum somatotropin and monensin on metabolism and production of periparturient Holstein dairy cows. J. Dairy Sci..

[54] Mutsvangwa T (2002). Effects of a monensin controlled-release capsule or premix on attenuation of subacute ruminal acidosis in dairy cows. J. Dairy Sci..

[55] Osborne J (2004). Effects of monensin on ruminal forage degradability and total tract diet digestibility in lactating dairy cows during grain-induced subacute ruminal acidosis. J. Dairy Sci..

[56] Erasmus L, Robinson P, Ahmadi A, Hinders R, Garrett J (2005). Influence of prepartum and postpartum supplementation of a yeast culture and monensin, or both, on ruminal fermentation and performance of multiparous dairy cows. Anim. Feed Sci. Technol..

[57] Eifert EDC (2005). Efeitos do fornecimento de monensina e óleo de soja na dieta sobre o desempenho de vacas leiteiras na fase inicial da lactação. Rev. Bras. Zootecnia.

[58] Van Vugt C (2005). Impact of monensin on methane production and performance of cows fed forage diets. Proc. N. Z. Soc. Anim. Prod..

[59] Bell J, Griinari J, Kennelly J (2006). Effect of safflower oil, flaxseed oil, monensin, and vitamin E on concentration of conjugated linoleic acid in bovine milk fat. J. Dairy Sci..

[60] Martineau R (2007). Effects of lasalocid or monensin supplementation on digestion, ruminal fermentation, blood metabolites, and milk production of lactating dairy cows. J. Dairy Sci..

[61] Yang W (2007). Effects of garlic and juniper berry essential oils on ruminal fermentation and on the site and extent of digestion in lactating cows. J. Dairy Sci..

[62] Karcher E, Pickett M, Varga G, Donkin S (2007). Effect of dietary carbohydrate and monensin on expression of gluconeogenic enzymes in liver of transition dairy cows. J. Dairy Sci..

[63] AlZahal O (2008). Effects of monensin and dietary soybean oil on milk fat percentage and milk fatty acid profile in lactating dairy cows. J. Dairy Sci..

[64] Grainger C (2008). Use of monensin controlled-release capsules to reduce methane emissions and improve milk production of dairy cows offered pasture supplemented with grain. J. Dairy Sci..

[65] Chung Y-H, Pickett M, Cassidy T, Varga G (2008). Effects of prepartum dietary carbohydrate source and monensin on periparturient metabolism and lactation in multiparous cows. J. Dairy Sci..

[66] Gehman A, Kononoff PJ, Mullins C, Janicek B (2008). Evaluation of nitrogen utilization and the effects of monensin in dairy cows fed brown midrib corn silage. J. Dairy Sci..

[67] Martinez C, Chung Y-H, Ishler V, Bailey K, Varga G (2009). Effects of dietary forage level and monensin on lactation performance, digestibility and fecal excretion of nutrients, and efficiency of feed nitrogen utilization of Holstein dairy cows. J. Dairy Sci..

[68] Petit HV (2009). The interaction of monensin and flaxseed hulls on ruminal and milk concentration of the mammalian lignan enterolactone in late-lactating dairy cows. J. Dairy Res..

[69] Fatahnia F, Roughani E, Hosseini A, Darmani KH, Zamiri M (2010). Effect of different levels of monensin in diets containing whole cottonseed on milk production and composition of lactating dairy cows. Iran. J. Vet. Res..

[70] Grainger C, Williams R, Eckard R, Hannah M (2010). A high dose of monensin does not reduce methane emissions of dairy cows offered pasture supplemented with grain. J. Dairy Sci..

[71] Hamilton SW, DePeters EJ, McGarvey JA, Lathrop J, Mitloehner FM (2010). Greenhouse gas, animal performance, and bacterial population structure responses to dietary monensin fed to dairy cows. J. Environ. Qual..

[72] Baumgard L (2011). Postabsorptive carbohydrate adaptations to heat stress and monensin supplementation in lactating Holstein cows. J. Dairy Sci..

[73] He M, Perfield K, Green H, Armentano L (2012). Effect of dietary fat blend enriched in oleic or linoleic acid and monensin supplementation on dairy cattle performance, milk fatty acid profiles, and milk fat depression. J. Dairy Sci..

[74] Abdi E, Fatahnia F, Banadaki MD, Azarfar A, Khatibjoo A (2013). Effects of soybeans roasting and monensin on milk production and composition and milk fatty acids profile of lactating dairy cows. Livest. Sci..

[75] Khodamoradi S (2013). Effect of monensin and vitamin E on milk production and composition of lactating dairy cows. J Anim. Physiol. Anim. Nutr..

[76] Rico D, Holloway A, Harvatine K (2014). Effect of monensin on recovery from diet-induced milk fat depression. J. Dairy Sci..

[77] Do Prado R, Côrtes C, Benchaar C, Petit H (2015). Interaction of sunflower oil with monensin on milk composition, milk fatty acid profile, digestion, and ruminal fermentation in dairy cows. Anim. Feed Sci. Technol..

[78] Hagen A, Martin R, Shaver R (2015). Effect of dietary monensin supplementation and amino acid balancing on lactation performance by dairy cows. Prof. Anim. Sci..

[79] Benchaar C (2016). Diet supplementation with cinnamon oil, cinnamaldehyde, or monensin does not reduce enteric methane production of dairy cows. Animal.

[80] Azarfar A, Satari Y, Kiani A, Khosarvinia H, Khaldari M (2016). Effects of monensin supplementation alone or in combination with Methafix on milk production and composition, ruminal parameters and serum metabolites of lactating dairy cows. Iran. J. Anim. Sci..

[81] Schären M (2017). Differential effects of monensin and a blend of essential oils on rumen microbiota composition of transition dairy cows. J. Dairy Sci..

