# Supplementary material for: Effects of monensin supplementation on lactation performance of dairy cows: a systematic review and dose–response meta‑analysis
Source: Sci Rep. 2023 Jan 11;13:568. doi: 10.1038/s41598-023-27395-9 (PMC9834228; doi:10.1038/s41598-023-27395-9)
Supplement: Supplementary file 1 — Supplementary Table S1. [file 41598_2023_27395_MOESM1_ESM.docx]

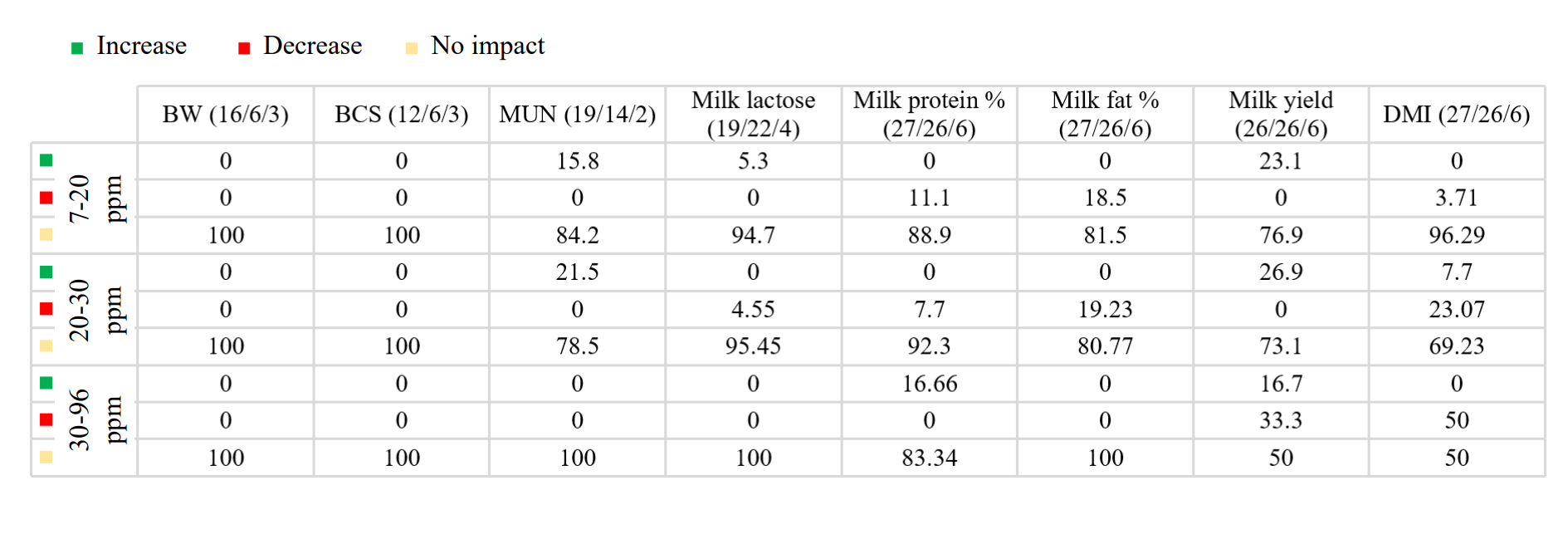


**Supplemental Table S1.** Percent effectiveness of "examined monensin doses" on outcomes in dairy cows. The effect was considered significant if the overall P value of the model was less than 0.05. Numbers in parentheses preceding results indicate the number of studies that examined the above dose ranges, separated by the slash '/'.
